# Combined effects of vitamin D and neferine on the progression and metastasis of colorectal cancer

**DOI:** 10.1007/s00432-022-04552-7

**Published:** 2023-01-26

**Authors:** Jinfeng Yang, Qinyu Zhang, Guanlin Huang, Jiacheng Cong, Ting Wang, Xiaoya Zhai, Juzheng Zhang, Guangying Qi, Lihua Zhou, Jiamin Jin

**Affiliations:** 1 School of Pharmacy, Guilin Medical University, Guilin, 541199 China; 2 Key Laboratory of Tumor Immunology and Microenvironmental Regulation, Guilin Medical University, Guilin, 541199 China; 3 Department of Immunology, Guilin Medical University, Guilin, 541199 China; 4 Guangxi Health Commission Key Laboratory of Tumor Immunology and Receptor-Targeted Drug Basic Research, Guilin Medical University, Guilin, 541199 China; 5 Department of Gastrointestinal Surgery, Affiliated Hospital of Guilin Medical University, Guilin, 541000 China

**Keywords:** Vitamin D_3_, Neferine, Colorectal cancer, Migration, Invasion

## Abstract

**Purpose:**

To investigate the synergistic effect of vitamin D and neferine on the growth and metastasis of colorectal cancer (CRC).

**Methods:**

The synergistic effect of biologically active form of vitamin D, VD_3_ and neferine on the treatment of CRC was investigated by bliss analysis. Colony formation and wound healing ability, migration and invasion ability, and epithelial mesenchymal transition of HCT-116 cells, as a response to the combination treatment with VD_3_ and neferine were evaluated.

**Results:**

VD_3_ and neferine showed a synergistic effect on CRC cell growth at a relatively low dose. The wound healing and colony formation capacity, cell migration and invasion abilities were all decreased by combination use of VD_3_ and neferine, compared to the VD_3_ or neferine treated single group. Furthermore, VD_3_ and neferine significantly decreased the expressions of N-cadherin, vimentin, snail, and slug in HCT-116 cells.

**Conclusion:**

These data suggest that neferine enhances the anticancer capability of VD_3_ and reduces the dose dependency of VD_3_. The combination of vitamin D with neferine appears to be a potential therapeutic strategy for CRC.

## Introduction

Colorectal cancer (CRC) is the third most common cancer in men and second in women, leading to a high incidence and mortality (Hull 2021). Nutritional imbalance and inappropriate dietary defects have been shown to be the main reasons for the development of CRC (Thanikachalam and Khan 2019). One potential strategy for the nutritional prevention of CRC is the use of supplements that could provide greater individual nutrient exposure than obtained through diet. Garland et al. in 1980, reported that a better vitamin D status in an individual could effectively decrease the risk of CRC development (Garland and Garland 2006).

Vitamin D, which is available mainly as D_2_ and D_3_, is a liposoluble nutraceutical vitamin (Meeker et al. 2016). Vitamin D is present in dietary sources, converted to 25-hydroxyvitamin D in the liver (Rinninella et al. 2022), and is further modified to 1ɑ, 25-dihydroxyvitamin D_3_ (VD_3_, also known as calcitriol), which is the biologically active form of vitamin D. (Yu et al. 2021) Furthermore, emerging studies have reported that vitamin D plays an important role in various cancers (Feldman et al. 2014). For example, a high amount of vitamin D intake was associated with a reduced rate of the early onset of CRC (Kim et al. 2021); VD_3_ could inhibit the migration and invasion of thyroid cancer cells (Chiang et al. 2015); VD_3_ influences cellular iron homeostasis, which further induces oxidative stress and death of breast cancer cells (Bajbouj et al. 2021). Furthermore, VD_3_ increases 5-fluorouracil cytotoxicity against CRC (Aslam et al. 2021). Vitamin D analogs combined with irinotecan or oxaliplatin could be used in CRC treatment (Milczarek et al. 2013). These findings support the notion that vitamin D exerts synergistic antitumor effects in CRC. However, the efficacy of vitamin D therapy is often reduced by side effects and drug resistance. Therefore, identification of a new product that can be applied together with vitamin D as a combinatorial strategy to reduce dose-induced side effects is urgently needed.

Neferine is found in the seed-embryos of *Nelumbo nucifera*. It is a major bisbenzylisoquinoline alkaloid (Marthandam Asokan et al. 2018) and has been shown to have a therapeutic effect in several cancers, its anticancer potential has been widely reported: neferine inhibits the proliferation and migration of human prostate cancer stem cells by suppressing the activation of the MAPK/JNK pathway (Erdogan and Turkekul 2020); neferine can increase anticancer drug chemosensitivities through downregulating the Bcl-2 and NF-κB signaling pathway in ROS-dependent manner in human renal cancer cells (Kim et al. 2022); neferine has shown to enhance thyroid cancer cell apoptosis, but inhibit its invasion and metastasis (Li et al. 2022); neferine induces apoptosis of CRC cells by augmenting intracellular uptake of Cisplatin (Manogaran et al. 2019a); combinatorial use of neferine with cisplatin exhibited stronger cytotoxic activity against cisplatin resistant colon stem cells compared to controls (Manogaran et al. 2022).

Increasing research has focused on the combination of natural dietary products and chemotherapeutic drugs in the treatment of CRC. This strategy is becoming the preferred therapeutic strategy against CRC. To our knowledge, there is no report that focuses on the combination effect of VD_3_ and neferine in CRC treatment. Therefore, our objective was to evaluate the potential effect and determine the underlying mechanism of VD_3_ in combination with neferine on the progression of CRC in the current study. Hence, the efficacy and applications of VD_3_ and neferine will provide a new therapeutic strategy to prevent drug resistance and adverse effects, which will further improve CRC therapy.

## Materials and methods

### Cells and compounds

Human colorectal cancer cell lines (HCT-116, SW480) and normal human colon mucosal epithelial cell lines (NCM460) were purchased from the American Tissue Culture and Preservation Center (ATCC). NCM460, HCT-116 and SW480 cells were cultured in Dulbecco modified Eagle medium (DMEM) complete medium supplemented with 10% fetal bovine serum (FBS) and 1% penicillin streptomycin (PS) at 37 °C and 5% CO_2._ Vitamin D_3_ (CAS No.:67-97-0) and neferine (CAS No.:2292–16-2) were purchased from Shanghai Macklin Biochemical Co., Ltd., China.

### Western blotting

Cells were lysed in ice cold RIPA buffer with protease inhibitor for 35 min, and centrifuged at 4 °C, 11,000 g for 15 min; 10 µg protein was subjected to SDS-PAGE and subsequently transferred to a polyvinyl difluoride membrane (Bio-Rad Laboratories, Inc., Hercules, CA, USA). The membranes were incubated with the primary antibody at 37 °C, for 1 h followed by incubation with the secondary antibody at 37 °C for 1 h. The primary antibodies were N-cadherin (Cat. no 22018-1-AP, Proteintech, USA), E-cadherin (Cat. no 60335–1-lg, Proteintech, USA), Vimentin (Cat. no 10366-1-lg, Proteintech, USA) Snail (Cat. no 13099-1-AP, Proteintech, USA), Slug (Cat. no 12129-1-AP, Proteintech, USA). Secondary antibodies were goat anti-mouse HRP (Cat. no SA00001-1, Proteintech, USA) or goat anti-rabbit HRP (Cat. no SA00001-2, Proteintech, USA). β-actin (Cat.no TA-09; Zsbio, China) was used as a housekeeping gene control.

### MTT assay

Approximately, 5 × 10^3^ NCM460, HCT-116, or SW480 cells were seeded to each well in a 96-well plate. Cells were incubated overnight for cell attachment. The cells were then treated with relative conditions of VD_3_ or neferine for 24 h; 10 μL of MTT (3-(4, 5-dimethylthiazol-2-Yl)-2, 5-diphenyl tetrazolium bromide—5 mg/ml) was added in medium 4 h before the end of the culture setting. Finally, 100 μL of dissolved solution (DMSO) was added, and the absorbance was detected by the spectrophotometer at 450 nm (Bio-Tek Instruments, VT). The combination of VD_3_ and neferine was analyzed according to the Chou–Talalay protocol with Calcusyn software (Biosoft).

### Colony formation assay

HCT-116 cells were treated accordingly. Cells were digested and seeded at 1 × 10^3^ cells / well in a 6-well plate. Cells were incubated in DMEM supplemented with 0.5% FBS and cultured under 5% CO_2_ at 37 °C for 15 days. At the end of cell culture, the medium was removed and the cells were immediately fixed with paraformaldehyde for 10 min at room temperature. The cells were then stained with 0.1% crystal violet (Sigma Aldrich, Germany) for 25 min and photographed by relative microscope.

### Wound healing assay

The treated cells were seeded in 6-well plates at a concentration of 8 × 10^5^/well. When the cells reached confluence, a 200 μL pipette tip was used to draw a straight wound in each well. Subsequently, the cell debris was removed. After that, cells were continued to be cultured in DMED supplemented with 2% FBS in the incubator. The wound closure or filling was evaluated using a microscope (Olympus, IX71) at a magnification of 40 × at the stated time points.

### Migration and invasion assays

Migration and invasion assays were performed according to the literature with slight modification. Briefly, Boyden chambers with Corning® Transwell polycarbonate membrane cell culture inserts (Sigma-Aldrich; Merck KGaA) were used as experimental carrier. Approximately 2 × 10^5^ cells were resuspended in 100 μL serum-free DMEM and seeded in the upper chamber, 500 μL DMEM containing 25% FBS was placed in the lower compartments. Cells were incubated under normal culture conditions for 24 h. At the end of the culture, the chamber was taken and migrated, or invaded cells were stained with 0.1% crystal violet solution and observed under microscope.

### Statistical analysis

All data were collected from 3 independent experiments under the same conditions. Data were analyzed using GraphPad Prism 8. Data were presented as mean ± standard deviation. The comparison of numerical data was analyzed using chi-square test and Spearman correlation analysis. The t test assay was used to compare the differences between two groups. *P < 0.05 represents a significant difference between the groups, ***P < 0.001 was extremely significant.

## Results

### Synergistic effect of VD_3_ and neferine on CRC cells

**Fig. 1:**
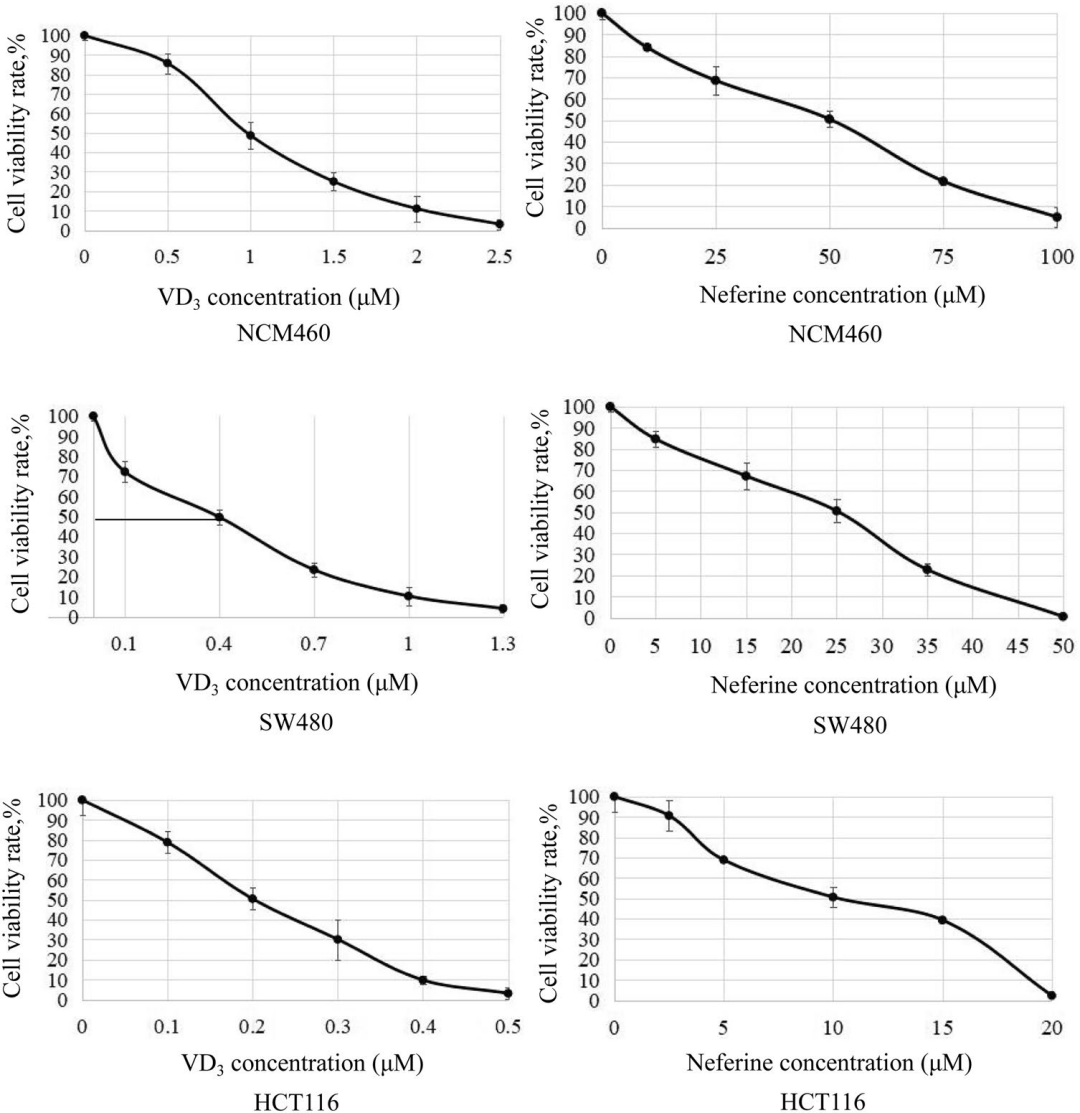
Effect of VD3 and neferine on the proliferation of CRC cells. Cell viability of NCM460, HCT-116, and SW480 cells was performed at different concentrations of VD_3_ or neferine. The IC50 value was determined by the MTT assay]. The experiments were conducted three independent times

To determine the synergistic effect of VD_3_ (Fig. 1, left) and neferine (Fig. 1, right) on CRC cells,^16^ NCM460, HCT-116, and SW480 cells were cultured with various concentrations of VD_3_ or neferine for 24 h. Cell proliferation was detected by the MTT assay. Both VD_3_ and neferine repressed viability of CRC cells in a dose-dependent pattern (Fig. 1). The IC_50_ values of VD_3_ in SW480 and HCT-116 cells were 0.4 μM and 0.2 μM, respectively; while the IC_50_ values of neferine in SW480 and HCT-116 cells were 25 μM and 10 μM, respectively (Table 1).

**Table 1:** IC_50_ of VD_3_ and Neferine on NCM460, SW480 and HCT-116 cells

Compound	Cell lines	IC_50_(μM)
VD_3_	NCM460	0.9
	SW480	0.4
	HCT-116	0.2
Neferine	NCM460	50
	SW480	25
	HCT-116	10

Next, we used Bliss analysis to check the synergistic effect of VD_3_ and neferine. HCT-116 cells were stimulated with a series concentration of VD_3_ (0.1, 0.2, 0.3, 0.4, 0.5 μM) and neferine (0, 5, 10, 15, 20, 25 μM) for various combinations.

**Fig. 2:**
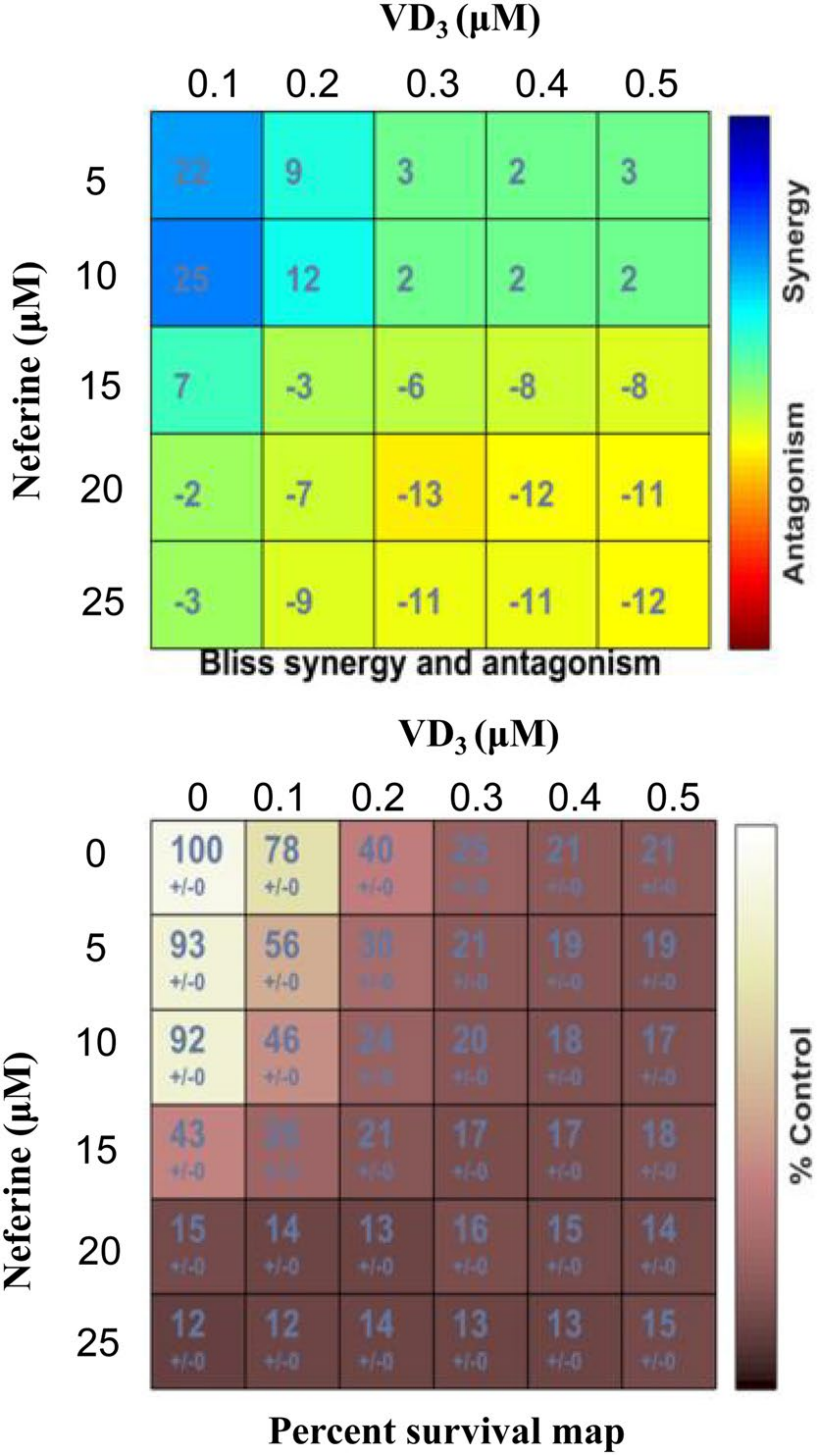
Synergistic effect of VD_3_ and neferine on CRC cells. Analysis of the synergistic effect of VD_3_ and neferine in HCT-116 cells. Positive bliss values indicate synergy. All experiments were conducted three times

As shown in Fig. 1c, the synergistic effect of VD_3_ and neferine on the HCT-116 cell was stronger than the antagonistic effect (Fig. 2). The combination regimens of VD_3_ (0.1 μM) and neferine (5 μM) substantially induced 50% cell death (Fig. 2). These data suggest that the combination use of VD_3_ and neferine significantly decreased the dose requirement for each substance compared to the other conditions. VD_3_ and neferine worked additively to induce CRC cell death. Thus, effective doses (0.1 μM for VD_3_ and 5 μM for neferine) were selected for subsequent assays.

### Combination of VD_3_ and neferine inhibit the colony formation, migration and invasion abilities of CRC cell

**Fig. 3:**
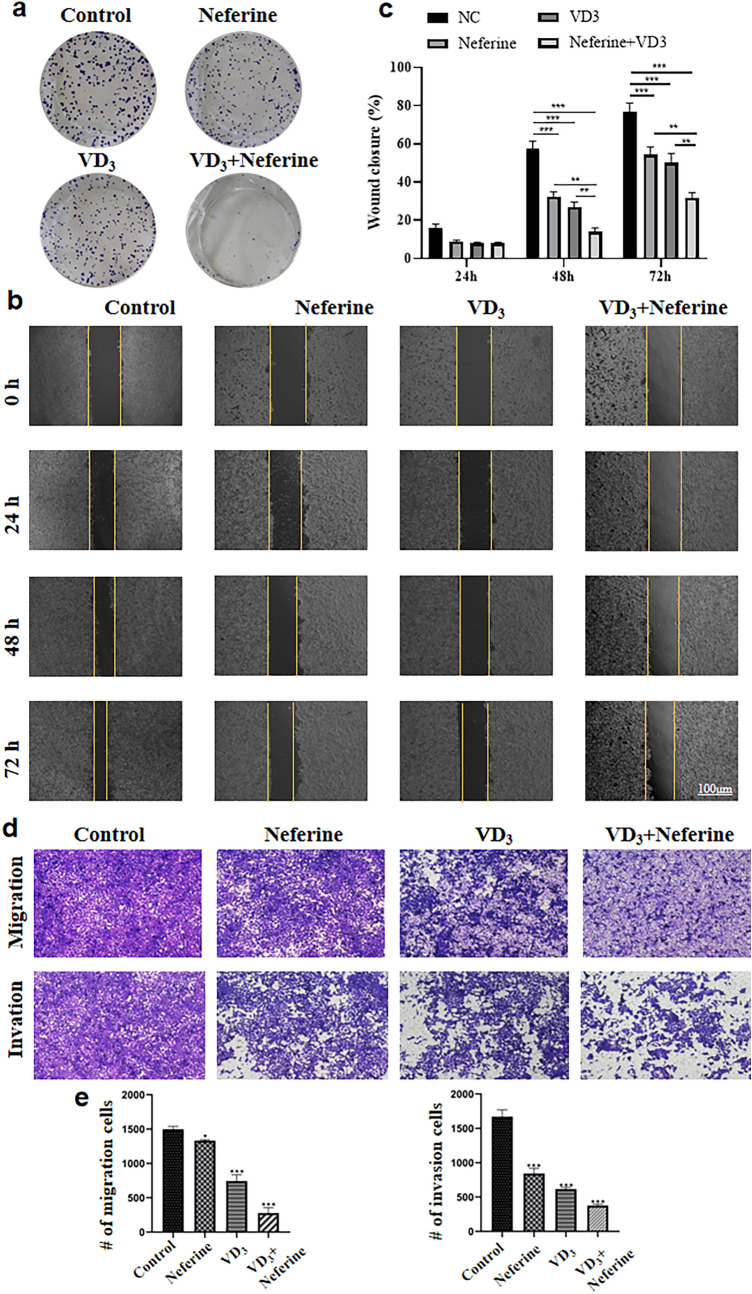
Combination effect of VD_3_ and neferine in CRC cell colony formation, migration and invasion abilities. **a** Colony formation assay of HCT-116 cells treated with VD_3_, neferine or a combination of VD_3_ and neferine. **b**,**c** Wound healing assay of HCT-116 cells treated with VD_3_, neferine or combination of VD_3_ and neferine. NC was set as 0. Magnification 200 × . **d**,**e** The migration (upper) and invasion (lower) abilities of cells treated with VD_3_, neferine or combination of VD_3_ and neferine was assessed by transwell assay. Magnification 200 × . All experiments were conducted three times. *P < 0.05; **P < 0.01; ***P < 0.001

To investigate the synergistic effect of VD_3_ and neferine on CRC cell growth, the ability to colonize and heal wounds of HCT-116 cells were examined. As shown in Fig. 3a, both VD_3_ (0.1 μM) and neferine (5 μM) reduced the colony formation of HCT-116 cells, while the combination of VD_3_ and neferine markedly inhibited the colony formation ability of HCT-116 cells. Next, we detected the effect of VD_3_ and neferine on the wound healing ability of HCT-116 cells. As expected, both VD_3_ and neferine showed a promised inhibitory effect on healing ability, while co-treatment with VD_3_ and neferine significantly dampened the wound healing ability of HCT-116 cell (Fig. 3b, c). These results indicated that combinatorial regimen treatments could better inhibit CRC cell growth.

We have shown that CRC cell growth was inhibited by the combination of VD_3_ and neferine. Next, we propose to further verify the synergistic effect of VD_3_ and neferine on CRC metastasis. The transwell assay exhibited that both VD_3_ and neferine alone can significantly decrease the number of migrated HCT-116 cells (Fig. 3d, e), while migrated HCT-116 cells cultured with VD_3_ and neferine together decreased significantly. In the invasion test, HCT-116 cells cultured with VD_3_ or neferine alone showed decreased invasion abilities, while a combination of VD_3_ and neferine could markedly reduce the number of invasion cells (Fig. 3d, e). These data indicate a significantly suppressive effect of VD_3_ and neferine on the migration and invasion abilities of CRC cells.

### Combination of VD_3_ and Neferine suppressed epithelial-mesenchymal transition of Colorectal Cancer Cell

**Fig. 4:**
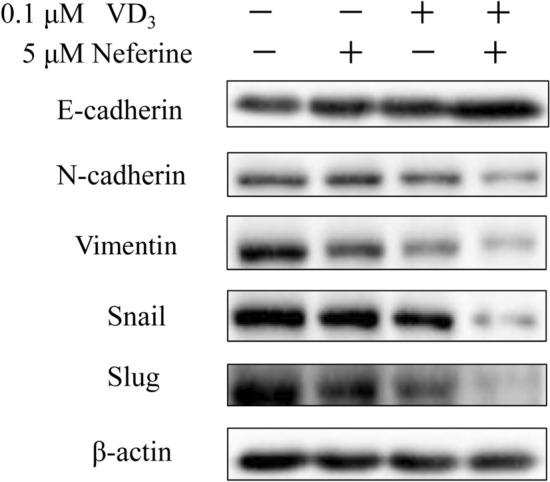
The combination of VD_3_ and neferine suppressed the epithelial-mesenchymal transition of CRC cell. Relative protein expressions of EMT of HCT-116 cells treated w/ith VD_3_, neferine, or a combination of VD_3_ and neferine were determined by Western blot. All experiments were conducted three times. *P < 0.05; ***P < 0.001

In ovarian cancer cells, vitamin D could suppress the epithelial–mesenchymal transition (EMT) by reducing Slug and Snail (Two EMT related proteins) and upregulation of E-cadherin.^19^ In the present study, we demonstrated that VD_3_ (0.1 μM) or neferine (5 μM) alone could slightly decrease EMT associated protein expression, while the combination of VD_3_ and neferine could significantly increase E-cadherin expression, while significantly decrease N-cadherin, vimentin, Snail and Slug expression by HCT-116 cells, compared to the other groups (Fig. 4). These data further demonstrated that the combinatorial regimen treatments could limit the EMT of HCT-116 cells.

## Discussion

In addition to the activity of vitamin D as a single agent, its anticancer activities in synergy with chemotherapeutic drugs have been attractive for several years. Combinatorial strategies, such as the use of phytoconstituents as adjuncts to vitamin D and relative approaches have become hotspots for cancer therapy. On the other hand, traditional medicinal herb have been widely accepted as new therapeutic agents in cancer research. Neferine is a bisbenzylisoquinoline alkaloid. It is the main component of the seed embryos of *N. nucifera* (Bharathi Priya et al. 2021). Neferine has been found to perform anticancer functions (Manogaran et al. 2019b). In the current study, we explored the synergistic effect of vitamin D and neferine on CRC cell growth and metastasis.

Various hallmarks of tumors, include proliferation, migration, invasion, and EMT. In the current study, we first aimed to optimize suitable working concentrations of VD_3_ and neferine in CRC cells. We set relative conditions for VD_3_ and neferine accordingly and determined the IC_50_ value of each substance in the CRC cell lines (HCT-116 and SW480) and healthy control (NCM460) (Fig. 1b). Next, using a Bliss synergy and antagonism assay, we confirmed there was a synergistic effect between VD_3_ and neferine. The percentage survival map assay further demonstrated that the combination of 0.1 μM of VD_3_ and 5 μM of neferine could reach the IC_50_ for cell growth (Fig. 2). These data indicated that the combination of VD_3_ and neferine could reduce the dose of each substance, which could further decrease its potential cytotoxicity on cells. Our data represents the first evaluation of the combination of vitamin D and neferine in CRC.

Next, we evaluated the impact of VD_3_ and neferine on CRC cell growth. According to previous findings, both VD_3_ and neferine showed inhibitory effects on HCT-116 cells at relative concentrations. To our surprise, the combination of VD_3_ and neferine exhibited significant cell growth inhibition, as detected by the colony formation assay (Fig. 3a) and the wound healing assay (Fig. 3b, c). These data strongly support our hypothesis that combination of VD_3_ and neferine has antiproliferative capability.

Cancer metastasis is one of the main causes of worse clinical outcome and survival in patients with CRC (Li and Chen 2011). Therefore, we performed a transwell setting to check the impact of VD_3_ and neferine on the migration, invasion and EMT abilities of CRC cells. As reported by other groups, VD_3_ alone can significantly inhibit the migration, invasion and relative EMT associate proteins in HCT-116 cells, while neferine alone showed a better inhibitory effect. In particular, the combination of VD_3_ and neferine together extremely dampened the invasion and migration abilities of HCT-116 cells (Fig. 3d, e). Furthermore, the expression of E-cadherin was increased, while the expression of N-cadherin, Vimentin, Snail, and Slug were strikingly downregulated by VD_3_ and neferine together (Fig. 4). These data demonstrated that the combination of VD_3_ and neferine could effectively inhibit CRC metastasis.

Collectively, we demonstrated that the combination of VD_3_ and neferine showed potential application in improving CRC cell growth and metastasis. The molecular mechanism of the benefit of the combination of VD_3_ and neferine may be similar to previously known mechanisms. Furthermore, the role of VD_3_ and neferine in the regulation of CRC cell metastasis could be an interesting field for future studies. Further research could focus on the antitumor effects of cotreatments with structural analogs of vitamin D and neferine for clinical application.

## Data Availability

The data sets generated during the current study are available from the corresponding author on reasonable requests.
